# Genome-Wide Identification and Characterization of the *Aux*/*IAA* Gene Family in Strawberry Species

**DOI:** 10.3390/plants13202940

**Published:** 2024-10-21

**Authors:** Xiaotong Jing, Quan Zou, Hui Yang

**Affiliations:** 1 Yangtze Delta Region Institute (Quzhou), University of Electronic Science and Technology of China, Quzhou 324003, China; 2018204005@njau.edu.cn (X.J.); zouquan@nclab.net (Q.Z.); 2 Institute of Fundamental and Frontier Sciences, University of Electronic Science and Technology of China, Chengdu 610054, China

**Keywords:** *Aux*/*IAA*, *Fragaria*, genome-wide identification, evolution, expression

## Abstract

Auxin is the first plant hormone found to play a dominant role in fruit growth, from fruit set to fruit ripening. Strawberry plants represent a suitable model for studying auxin’s biosynthesis, sensing, and signaling machinery. *Aux*/*IAA* genes are a classical rapid auxin-responsive family. However, the *Aux*/*IAA* gene family in *Fragaria* genus is poorly understood. In this study, a total of 287 *Aux*/*IAA* genes were identified in the eight strawberry genomes. Their physicochemical properties, domain structure, and cis-regulatory elements revealed the functional multiplicity of the strawberry *Aux*/*IAA*s. We used a phylogenetic analysis to classify these genes into 12 classes. In addition, based on synteny analysis, gene duplications, and calculation of the Ka/Ks ratio, we found that segmental duplications promote the evolution of *Aux*/*IAA*s in *Fragaria* species, which is followed by purifying selection. Furthermore, the expression pattern and protein–protein interaction network of these genes in *Fragaria vesca* revealed various tissue-specific expressions and probable regulatory functions. Taken together, these results provide basic genomic information and a functional analysis of these genes, which will serve to expand our understanding of the direction in which the *Aux*/*IAA* gene family is evolving in *Fragaria* species.

## 1. Introduction

Fruit development is a complex process involving multiple phytohormones, within which auxin was the first plant hormone to be discovered. Auxins play a critical role in tuning fruit growth, from fruit set to fruit ripening [1]. Auxins mainly exert their effects by coordinating auxin synthesis and metabolism [2,3], polar transport [4], and signal transduction [5] pathways. Through the classical signal transduction pathways, auxin facilitates auxin response factor (ARF) transcriptional activity by eliciting the degradation of the auxin/indole-3-acetic acid (*Aux*/*IAA*) transcription repressors [6].

*Aux*/*IAA* genes belong to a classical rapid auxin-responsive family. They encode short-lived nuclear proteins, which have half-lives as short as 6–8 min in pea plants (8 min for *PSIAA4* and 6 min for *PSIAA6*) [7], 10–60 min in *Arabidopsis* (10 min for *AtIAA7*/*AtIAA17* and 60 min for *AtIAA28*) [8], and 11–120 min in maize (11 min for *ZmIAA2* and 120 min for *ZmIAA15*) [9]. Their half-lives are determined by their domain II. The genome of *Arabidopsis thaliana* has 29 genes encoding Aux/IAA proteins, with the majority of these genes exhibiting four conserved domains (domains I–IV) [10]. Each domain contributes to a different function. Among them are domain I, which bears a Leu-rich motif (LxLxLx, where L is Leu and x is one of several different amino acids) and is responsible for the repression of proteins [11], and domain II, which contains a conserved GWPPV degron in nearly all *Arabidopsis* Aux/IAA and directly interacts with the SCF^TIR1^ complex. This interaction leads to the polyubiquitination and degradation of Aux/IAA proteins [12]; The homodimerization and heterodimerization of ARFs and the Aux/IAAs are induced by domains III and IV by way of the Phox and Bem1p (PB1) domain [13]. Until now, studies about mutations in *Aux*/*IAA*s have revealed that this family has distinct functions within a range of developmental processes, including floral organ development, embryo development, lateral root elongation, phototropisms, and so on [14]. The *Aux*/*IAA* family has been recognized and studied in various flowering plants, such as the crop plants rice [15] and maize [16]; the vegetable species *Solanum* [17,18] and *Brassica* [19,20,21]; the fruits papaya [22] and apple [23]; the tree species *Populus trichocarpa* [24], *Carya cathayensis* [25], *Acer rubrum* [26], *Populus simonii* [27], and *Paulownia fortune* [28]; the forage grasses orchardgrass [29] and alfalfa [30]; the medicinal plants *Bletilla striata* [31], *Dendrobium ofcinale* [32], *Panax ginseng* [33], and *Artemisia argyi* [34]; and bamboo species [35,36].

Strawberry has long been used as an excellent model through which to study the molecular role of the hormone auxin in fruit growth and ripening [37,38]. Achenes, located on the outside of the receptacle, are rich sources of auxin [39]. When part or all of the achenes are removed, the fleshy fruit will fail to develop and ripen or will grow into an abnormal shape [37,40]. Exogenous auxin treatment may serve as a substitute for achenes and can cause horizontal and vertical expansion of fleshy fruit [38,41]. Therefore, the strawberry plant is a suitable means of studying auxin’s biosynthesis, sensing, and signaling machinery. Recent studies have indicated that ARF, Aux/IAA, and DELLA proteins interact with one another and play roles in regulating auxin signaling pathways in strawberry fruit growth [42]. In the *Fragaria* species, *Aux*/*IAA* genes have only been reported in *F. × ananassa and F. vesca*. For example, *FaAux*/*IAA1* and *FaAux*/*IAA2* transcripts may be involved in the early development of fruit, and their levels are observed to be significantly elevated in the early development stages, after which they decrease sharply in the ripening stage [43]. Additionally, a downward trend has been observed in the expression of *FvIAA4* following fertilization, which may negatively impact fruit initiation [44]. The *Fragaria* genus consists of around 25 species with varying ploidy levels, ranging from diploid (2×) to decaploid (10×) [45]. Among them, *F. × ananassa* is a young cultivated species, which is an allo-octoploid (8×) and originated from natural crosses between *F. virginiana* (8×) and *F. chiloensis* (8×). Diploid strawberry plants, such as *F. bucharica*, *F. chinensis*, *F. daltoniana*, *F. hayatai*, *F. iinumae*, *F. mandshurica*, *F. nilgerrensis*, *F. nipponica*, *F. nubicola*, *F. pentaphylla*, *F. vesca*, and *F. viridis*, etc., are the most abundant of all *Fragaria* [45]. These wild diploids exhibit varied morphology and physiology. *F. vesca* was chosen as the genomic reference for *Fragaria* thanks to its many advantages, including its rapid generation time and ability to easily propagate [46]. It differs from *F. vesca*, *Fragaria* genus including *F. bucharica*, *F. chinensis*, *F. mandshurica*, *F. nipponica*, *F. nubicola*, *F. pentaphylla*, and *F. viridis*, which are self-incompatible [45]. Mature fruits of *F. viridis* have an apple-like aroma [47], while *F. nilgerrensis* fruits have peach-like and banana-like aromas [48]. Among these diploid strawberries, *F. iinumae*, *F. nipponica*, *F. vesca*, and *F. viridis* might contribute to the subgenomes of *F. × ananassa* [49].

For the purpose of understanding the role of *Aux*/*IAA*s in the growth of strawberry fruits, we have comprehensively identified the *Aux*/*IAA*s in the octoploid strawberry as well as the extant relatives of each diploid progenitor species. We also examined the sequence structure characteristics of these genes in various species. Additionally, we explored the evolutionary trajectory and expansion mechanisms of *Aux*/*IAA*s in *Fragaria* based on interspecific collinearity. Moreover, the tissue expression patterns and protein–protein interaction (PPI) networks were investigated in the *Aux*/*IAA* genes. To sum up, out study will contribute to better annotation of *Fragaria* spp. genomes, including *F. iinumae*, *F. mandshurica*, *F. nilgerrensis*, *F. nipponica*, and *F. viridis*, and provide insights into the fruit development process with the aim of increasing crop production and fruit quality.

## 2. Results

### 2.1. Identification and Characterization of Aux/IAAs in Fragaria

To explore the evolutionary histories of the *Aux*/*IAA* family in the various ploidy strawberries, we identified the putative *Aux*/*IAA* genes from six diploid strawberries and two octoploid strawberries using the BLAST [50] and HMMER [51] search tools. Then, the supposed *Aux*/*IAA* genes were further confirmed to contain a conserved AUX_IAA (PF02309) domain in accordance with the Pfam database [52] and NCBI’s Conserved Domain Database [53]. After filtering out the genes found to possess B3 and ARF domains, thereby sustaining the auxin response factor (ARF), 287 *Aux*/*IAA*s were identified in the eight strawberry genomes. They were named according to the phylogenetic relationship between strawberries and *Arabidopsis*. Among these strawberry species, *F. × ananassa* has the largest *Aux*/*IAA* family (82 members), and the fewest (18) members were found in *F. iinumae*. Table S1 contains detailed information about these genes, such as gene ID, chromosome location, and sequence length. The length of strawberry *Aux*/*IAA* coding sequences (CDSs) varied from 309 bp for *FaIAA11b* to 1221 bp for *FveIAA12*. In addition, strawberry Aux/IAA protein’s physical and chemical properties were analyzed using the Expasy-ProtParam tool (https://web.expasy.org/protparam/) (accessed on 19 April 2024) in this study (Table S2). The molecular weight of those proteins ranged from 11.76 to 43.92 kD, and the theoretical isoelectric point ranged from 4.81 to 9.54. Furthermore, most Aux/IAA members (87.11%, 250/287) were unstable (instability index > 40), and all members were hydrophilic proteins (GRAVY index < 0).

### 2.2. Molecular Structure of Aux/IAA Proteins in Fragaria

**Figure 1:**
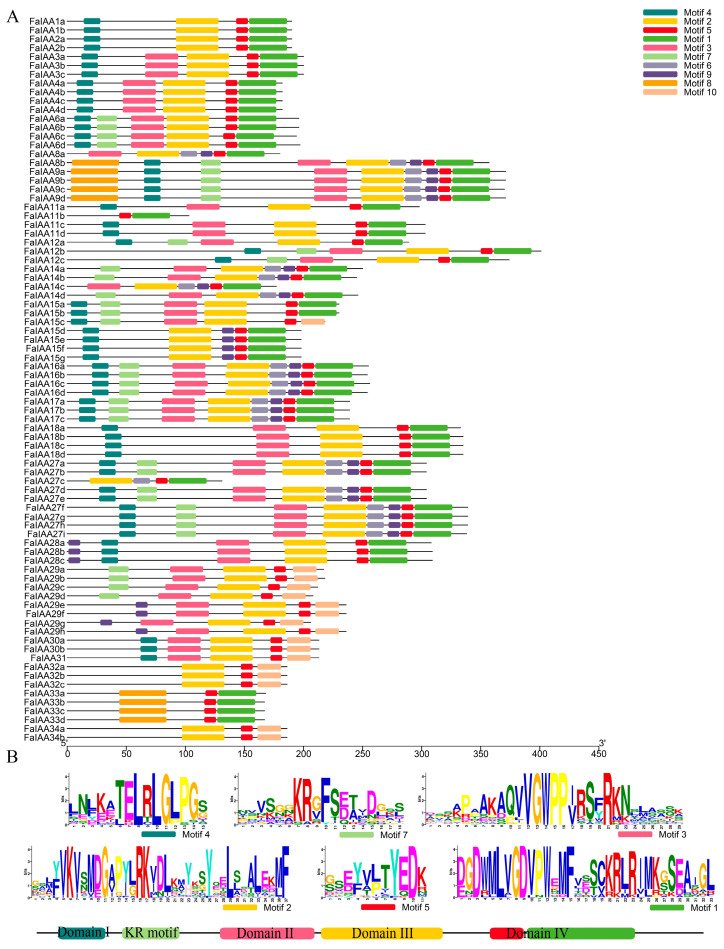
Motif analysis of the Aux/IAAs in *F. × ananassa*. All schemes follow the same formatting. (**A**) Conserved motifs of FaIAA proteins; (**B**) sequence logos of six conserved motifs in *F. × ananassa*.

To explore the conservation of these Aux/IAAs, we investigated the conserved motifs using the MEME Suite (https://meme-suite.org/meme/) (accessed on 30 April 2024), which elucidated the diversity and comparability of the Aux/IAAs. In total, 10 conserved motifs were found in the Aux/IAAs of *Fragaria*, namely, motifs 1–10 (Figure 1, Figures S1 and S2). Among them, five different conserved motifs, motifs 4, 2, 5, 1, and 3, were mapped to the most Aux/IAA proteins (Figure 1A). Among them, motif 4 has a classical “LxLxLx” motif, using which we identified domain I (Figure 1B), which was missing from the members of IAA14, IAA29, IAA32, IAA33, IAA34, and IAA12 of *F. mandshurica* (Figure S2D). Motif 2 corresponded to domain III, which was missing from the members of IAA33. All strawberry Aux/IAA proteins contained motif 5, with the exception of FcIAA15c, FiIAA3, and two Aux/IAA16 members, FiIAA16 and FnlIAA16. Motif 5 was included in domain IV. In addition, motif 1 was also included in domain IV, the conserved “GDVP” of which may contribute to electrostatic protein interactions [54]. We found that this function potentially was missing from the IAA15c, IAA29, IAA30, IAA31, IAA32 and IAA34 members, IAA16 of *F. iinumae*; IAA14 of *F. mandshurica*; and IAA17 and IAA20 of *F. nilgerrensis* (Figure S2). Motif 3, with a classical “GWPPV” motif, corresponded to domain II, which plays a role in rapid protein turnover. IAA1, IAA2, IAA32, IAA33, IAA34, and some IAA15 members may lack this function. Furthermore, motif 7 has a conserved “KR” motif located between domain I and domain II, and is highly conserved in the proteins of IAA6, IAA8, IAA9, IAA14, IAA17, IAA27, and IAA29. Interestingly, in this work, we found that motif 8 was observed in the Aux/IAAs, including IAA8, IAA9, and IAA33 members, with the exception of the two octoploid IAA8 members (FaIAA8a and FcIAA8a). The functions of this motif have not yet been reported. Moreover, we also measured the integrity of the Aux/IAAs in *Fragaria*. This result indicated that diploid Aux/IAA proteins are more complete than octoploid proteins (Table 1).

### 2.3. Evolutionary Tree Analysis and Classification of the Aux/IAA Gene Family in Fragaria

**Figure 2:**
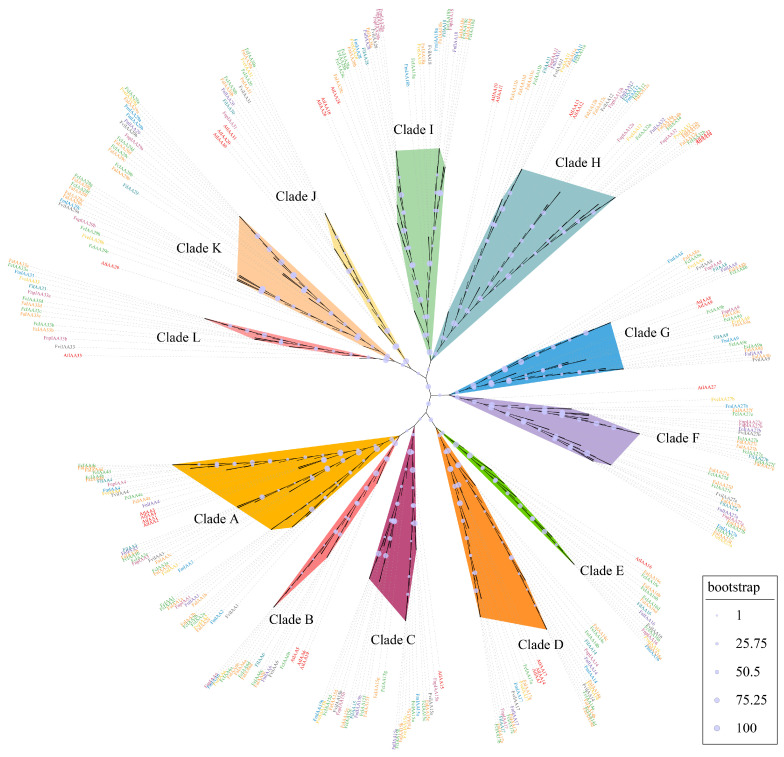
Unrooted maximum-likelihood tree analysis of *Aux*/*IAA*s. *Aux*/*IAA*s were classified into 12 clades (A–L). As shown in the legend at bottom right, bootstrap values are represented by different sizes of bubbles.

To probe into the evolutionary relationships among the *Aux*/*IAA*s from six diploid strawberries (*F. iinumae*, *F. mandshurica*, *F. nilgerrensis*, *F. nipponica*, *F. vesca*, and *F. viridis*), two octoploid strawberries (*F. × ananassa* and *F. chiloensis*), and *A. thaliana*, a maximum-likelihood tree was produced by MEGA11 [55] and based on the 316 Aux/IAA protein’s amino acid sequences, including 29 members of *A. thaliana* and 287 members of *Fragaria* spp. Based on the evolutionary tree, these Aux/IAAs were classified into 12 major clades: IAA1/2/3/4 (Clade A), IAA5/6/19 (Clade B), IAA15 (Clade C), IAA14/17 (Clade D), IAA16 (Clade E), IAA27 (Clade F), IAA8/9 (Clade G), IAA10/11/12/13/32/34 (Clade H), IAA18/28 (Clade I), IAA20/30/31 (Clade J), IAA29 (Clade K), and IAA33 (Clade L) (Figure 2).

### 2.4. Synteny and Ka/Ks Analysis of Aux/IAAs in Fragaria

**Figure 3:**
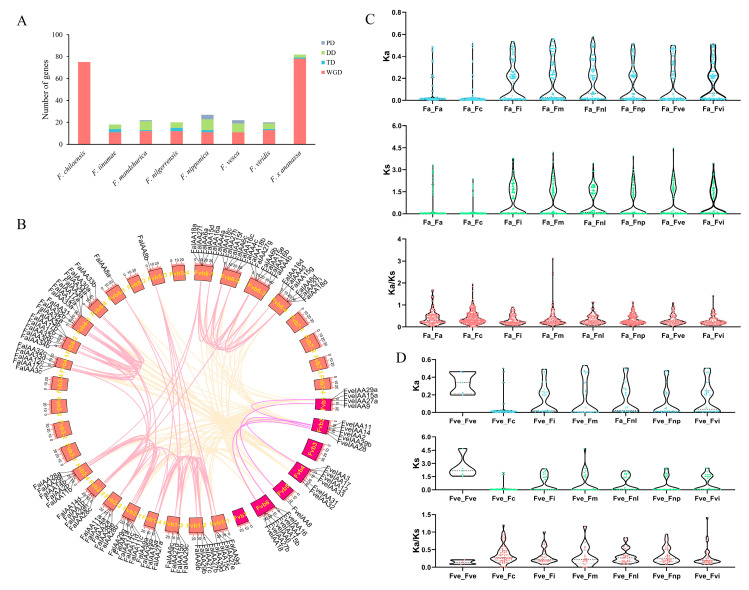
Synteny and KaKs analysis of *Aux*/*IAA* genes in strawberries. (**A**) Gene duplication types of *Aux*/*IAA*s. (**B**) Chromosomal collinearity relationships between *F. vesca* and *F. × ananassa*. *F. × ananassa* and *F. vesca* chromosomal regions are colored salmon and violet, respectively, and different-colored lines represent orthologous or paralogous gene pairs. (**C**) The paralogous *Aux*/*IAA* gene pairs in *F. × ananassa*, and orthologous *Aux*/*IAA* gene pairs between *F. × ananassa* and seven *Fragaria* species (including *F. chiloensis*, *F. iinumae*, *F. mandshurica*, *F. nilgerrensis*, *F. nipponica*, *F. vesca*, and *F. viridis*) were measured for Ka, Ks, and Ka/Ks. (**D**) Ka, Ks, and Ka/Ks values of paralogous *Aux*/*IAA* gene pairs in *F. vesca* and orthologous gene pairs between *F. vesca* and six *Fragaria* species (*F. chiloensis*, *F. iinumae*, *F. mandshurica*, *F. nilgerrensis*, *F. nipponica*, and *F. viridis*).

A collinearity analysis was carried out using Tbtools-II [56] ao that we might further understand the evolutionary relationships among *Fragaria Aux*/*IAA* genes. We explored the distribution of duplicate events in eight strawberry species. Among the 286 genes (with the exception of *FviIAA29b*), we identified instances of 223 whole-genome duplication (WGD), 43 of dispersed duplication, 11 of tandem duplication, and 9 of proximal duplication events (Table S1). Octoploid strawberries had more WGD events involving *Aux*/*IAA* family genes than diploid strawberries. Notably, all *Aux*/*IAA*s of *F. chiloensis* expanded through the WGD, while *Aux*/*IAA*s in diploid strawberries expanded mainly through WGD and dispersed duplication (Figure 3A). Additionally, based on the collinearity analysis, 2309 gene pairs, including 8 gene pairs formed through tandem duplication, were found in the strawberry *Aux*/*IAA*s. Among them, we identified 5 and 120 gene pairs produced by segment duplications in *F. vesca* and *F. × ananassa*, respectively. Additionally, 103 gene pairs experienced segment duplications between the *F. vesca* and *F. × ananassa* (Figure 3B). We further calculated the values of Ka and Ks of all gene pairs with the KaKs_Calculator [57] (Figure 3C,D and Figure S3). The findings indicated that all gene pairs exhibited Ka < 1, and 71.50% (1651/2309) of gene pairs exhibited Ks < 1. The Ka/Ks distribution of the gene pairs showed that most had Ka/Ks < 1, implying that the *Aux*/*IAA* gene family may largely have undergone purifying selection during the evolutionary process. Moreover, a high value of Ka/Ks (>1) was detected in 45 gene pairs, including the members of *IAA3* (5 pairs), *IAA6* (6 pairs), *IAA18* (5 pairs), *IAA29* (7 pairs), *IAA32*/*34* (7 pairs), etc. (Figure S4, Table S3). Among them, three gene pairs—*FaIAA29c*-*FmIAA29b*, *FaIAA29d*-*FmIAA29b*, and *FcIAA29c*-*FmIAA29b*—exhibited Ka/Ks > 2, indicating strong positive selection of these genes.

### 2.5. Predicting the Promoter Cis-Acting Elements of Aux/IAAs

**Figure 4:**
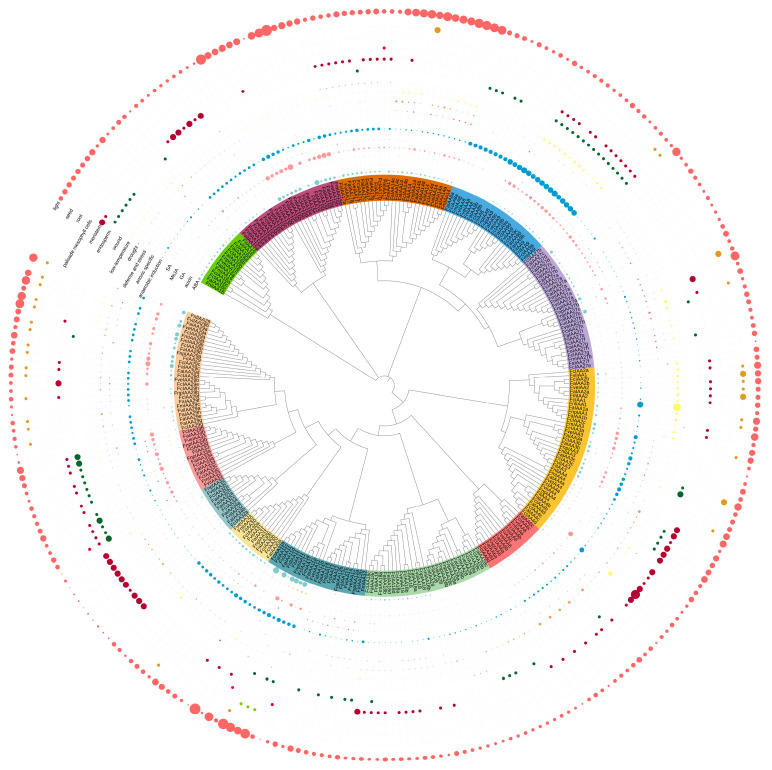
Promoter cis-acting elements predicted in the *Aux*/*IAA* family of *Fragaria*. As shown in the upper-left corner of the figure, four types of cis-acting elements were found: hormone response cis-acting elements (ABA, auxin, GA, MeJA, and SA), stress response cis-acting elements (anaerobic induction, anoxic-specific, defense and stress, drought, low temperature, and wound), tissue development-related cis-acting elements (endosperm, meristem, palisade mesophyll cells, root, and seed), and light-responsive cis-acting elements. Bubbles with various sizes and colors indicate the number of different types of cis-acting elements.

To detect the feasible regulatory modes of the *Aux*/*IAA*s, we analyzed the cis-acting element located within the 2000 bp sequences preceding the translation start sites of genes in the PlantCARE database (http://bioinformatics.psb.ugent.be/webtools/Plantcare/html/) (accessed on 9 May 2024). Then, we mainly analyzed and screened four types of cis-acting elements, which were largely hormone response elements, stress response elements, tissue development-related elements, and light-responsive elements (Figure 4). Five hormone response elements were found: abscisic acid (ABA), auxin, gibberellin acid (GA), methyljasmonate (MeJA), and salicylic acid (SA). Among them, 81.47% (233/286), 47.90% (137/286), 41.26% (118/286), 68.18% (195/286), and 43.71% (125/286) promoters of the *Aux*/*IAA* family carry ABA-, auxin-, GA-, MeJA-, and SA-responsive elements in strawberries, respectively. The promoters of the *IAA11* subfamily contain many ABA- and auxin-responsive elements. Half of the members of the *IAA15* and *IAA29* subfamilies contain response elements of ABA and MeJA, respectively. We also discovered six crucial stress-responsive regulatory elements in strawberry *Aux*/*IAA* promoter sequences: anaerobic induction (95.45%, 273/286), anoxic-specific (6.99%, 20/286), defense and stress (29.37%, 84/286), drought (54.90%, 157/286), low temperature (32.87%, 94/286), and wound (6.99%, 20/286). The *IAA8* and *IAA9* subfamilies contain up to six anaerobic induction elements. *FcIAA1* not only contains many anaerobic induction elements but also harbors the most low-temperature-responsive elements. Moreover, some tissue development-related elements were observed, such as the endosperm (64 sequences), meristem (105 sequences), palisade mesophyll cells (4 sequences), root (3 sequences), and seed (32 sequences). Among them, the subfamilies of *IAA4* and *IAA20*/*30*/*31* show many meristem expression elements, and the *IAA33* subfamily has many endosperm expression elements. Of the *IAA11* members, *FmIAA11*, *FcIAA11a*, and *FaIAA11a*, each contain a root-specific regulatory element. In addition, all the strawberry IAA promoter sequences contain a number of light-responsive elements, ranging from 2 (*FnpIAA32*, *FcIAA32b*, and *FaIAA32c*) to 26 (*FviIAA11*, and *FcIAA15f*). We found that half of the *IAA15* subfamily contain many ABA-, MeJA-, and light-responsive elements, and the other half contain many meristem expression elements. Altogether, an analysis was conducted on the cis-acting elements that will enhance our understanding of the roles of the *Aux*/*IAA*s in *Fragaria*.

### 2.6. Aux/IAA Genes’ Expression Profiles in Different Tissues of F. vesca

**Figure 5:**
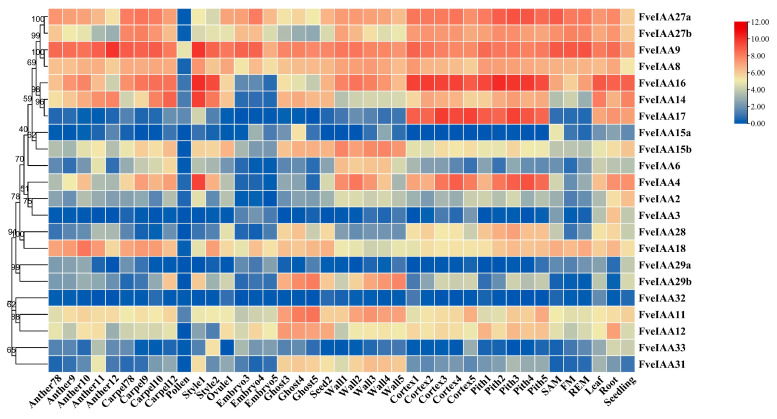
*FveIAA* gene tree and expression profiles of *Aux*/*IAA*s in *F. vesca*. SAM, FM, and REM represent the shoot apical meristem, flower meristem, and receptacle meristem, respectively.

*Aux*/*IAA* family members have distinct roles to play in plant growth and development processes. In this work, their expression patterns were studied in the development of the reproductive and vegetative tissues of the strawberry model *F. vesca* based on the annotation of the *F. vesca* v4 genome [58]. As shown in Figure 5, *FveIAA9* was highly expressed in all tissues and abundantly expressed in the anther 12 and style 1 stages. *FveIAA17* showed high expression in the cortex and pith development stages. *FveIAA4* and *FveIAA14* were significantly expressed in the style 1 stage. Additionally, we found that all *Aux*/*IAA* members showed a low degree of expression in pollen. In the seed development process, *FveIAA11*, *FveIAA15b*, *FveIAA16*, *FveIAA29b*, and *FveIAA31* largely accumulated in the ghost and wall development stages; *FveIAA8*, *FveIAA9*, and two *FveIAA27s* (*FveIAA27a*/*b*) were present at high levels in the embryo development process. Unlike *FveIAA29b*, *FveIAA29a* was weakly expressed in all tissues, as was *FveIAA32*. In addition, many *Aux*/*IAA*s were strongly expressed in vegetative tissues, including leaves, roots, and seedlings.

### 2.7. Analysis of Aux/IAA Protein–Protein Interaction Network

**Figure 6:**
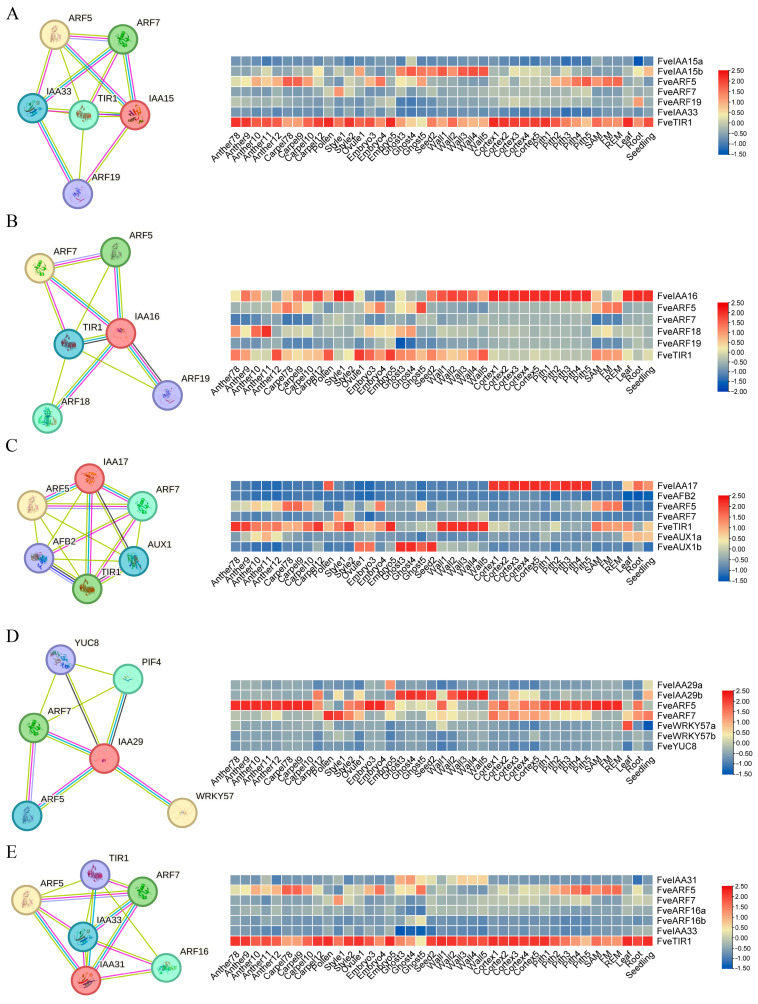
Five PPI networks and tissue expression patterns of *Aux*/*IAA*s and related hub genes. (**A**) PPI networks and tissue-specific expression profile of *IAA15* and related hub genes; (**B**) expression profile of *IAA16* and related hub genes from the PPI network; (**C**) *IAA17*-related hub genes were selected from the PPI network and their expression levels were analyzed; (**D**) PPI network and gene expression patterns analysis of *IAA29* and related hub genes; (**E**) PPI network and expression profile of *IAA31* and hub genes.

We identified five PPI networks for the proteins encoded by specific expression genes, including three ghost-and-wall-specific expression genes (*FveIAA15b*, *FveIAA29b*, and *FveIAA31*) and two cortex-and-pith-specific expression genes (*FveIAA16* and *FveIAA17*), which were established based on the known interactions of *A. thaliana* homologues on the STRING online website (http://cn.string-db.org) (accessed on 7 August 2024) [59,60,61,62] (Figure 6). The majority of interactions occurred between Aux/IAA and ARF proteins. For example, ARF5 and ARF7 were predicted to interact with five Aux/IAA proteins. Interestingly, we found that *FveARF5* had an expression pattern that opposed that of the five *IAA* genes in *F. vesca* (Figure 6). In addition, it was anticipated that Aux/IAA proteins would engage with other auxin signaling proteins; for instance, five Aux/IAAs (with the exception of IAA29b) also interacted with TIR1. Unlike *FveIAA16* and *FveIAA17*, *FveTIR1* was minimally expressed in cortex and pith tissues (Figure 6B,C). IAA17 was also predicted to interact with AFB2 and AUX1, while IAA29 was predicted to interact with YUC8 (Figure 6C,E). Moreover, IAA29 was also predicted to interact with WRKY57, which showed leaf-specific expression in the strawberry (Figure 6E).

## 3. Discussion

*Aux*/*IAA*s act as pivotal factors that regulate the expression of downstream transcription factors in auxin signaling transduction. Following the ongoing advancements in whole-genome sequencing technology, *Aux*/*IAA*s of several species have been identified [14,20]. Among these plant species, the number of *Aux*/*IAA*s varies significantly and ranges from 1 in *Marchantia polymopha* [63] to 119 in *Brassica napus* [21]. Based on different tree construction methods and numbers of sequences, the *Aux*/*IAA* family is divided into different clades in different plants. In *Populus simonii*, 33 *PsIAAs* were categorized into three subgroups using the neighbor-joining (NJ) approach [27]: all 149 Aux/IAA proteins—including *Arabidpsis*, rice, and ginseng *Aux*/*IAA* family members—were classified into six groups through the maximum-likelihood method using the DCMut + F+ R4 model [33]. To unveil the origins of the *Aux/IAAs*, Wu et al. [64] used 253 canonical *Aux/IAA* members from eudicots, grasses, amborellales, gymnosperms, pteridophytes, and bryophytes to analyze evolutionary relationships under the assumption that the auxin response pathway mediated by *Aux*/*IAA*s first emerged in land plants. To investigate the evolution of the *Aux*/*IAA*s in greater depth, all *Aux*/*IAA* genes from 406 species were divided into groups I–VIII. The results indicated that charophytes were clustered into group VI, and basal angiosperms were clustered into all subgroups except group I [65]. In our study, after further division, the *Aux*/*IAA* genes of eight strawberries were classified into 12 major clades. This result showed phylogenetic relationships of the *Aux*/*IAA* gene family between the different strawberry species, but could not comprehensively represent *Aux*/*IAA*s’ evolution in the *Fragaria* genus in the *Rosaceae* family, and this topic will require further exploration.

Additionally, a large proportion of *Aux*/*IAA*s expanded over the course of their evolution via both segmental and tandem duplications in the plants, among which segmental duplications showed a greater effect [14]. In *A. thaliana*, 75.86% of *Aux*/*IAA*s were found to be duplicated via segmental duplication events [64]. Among 63 soybean *Aux*/*IAA*s, 90.5% were from segmental duplications [66]. In this study, we found that diploid and octoploid strawberries of the *Aux*/*IAA* family have presumably undergone dissimilar forms of expansion. Some 40~65% of diploid *Aux*/*IAA*s originated from segmental duplications, while 95.12% and 100% of *Aux*/*IAA*s were derived from segmental duplications in *F. × ananassa* and *F. chiloensis*, respectively. *F. × ananassa*, the cultivated strawberry, is a young domesticated plant. It stems from natural hybrids of *F. virginiana* and *F. chiloensis* in Europe [67]. Among other taxa, flowering plants have experienced continuous whole-genome duplication, resulting in genomes made up of several homologous subgenomes [68]. Many scholars have put forward that four subgenomes—*F. iinumae*, *F. nipponica*, *F. vesca*, and *F. viridis*—are the subgenome progenitors of *F. × ananassa* [49,69]. Therefore, we identified gene pairs between *F. × ananassa* and its four subgenomes and found that segmental duplications drove *Aux*/*IAA* evolution in *Fragaria* species, which was followed by purifying selection: their evolution has been conservative (Figure 3C). This result is similar to findings in *Panax ginseng* [33]. Evolution of protein sequences is influenced by the constraint of purifying selection or the fixation of positive selection at the molecular evolution level [70]. Ka/Ks > 1 is considered the signature of positive selection [71]. In this work, 45 gene pairs were acquired based on intraspecific and interspecific collinearity analyses. Additionally, the gene pairs displayed Ka/Ks values ranging from 1.01 (*FveIAA4*-*FiIAA4*) to 3.13 (*FaIAA29d*-*FmIAA29b*) (Table S3). In *P. ginseng*, two gene pairs—*PgIAA25*-*PgIAA75* (homologue *AtIAA19*) and *PgIAA14*-*PgIAA40* (homologue *AtIAA30*/*31*)—underwent positive selection throughout their evolutionary history [33]. Similarly, *FaIAA31*-*FcIAA30a* also showed Ka/Ks values of >1 in this study (Table S3). We also found that *IAA8*, *IAA9*, *IAA14*, and *IAA27* members expanded at a Ka/Ks value higher than that of strawberries and other plant species. For instance, one gene pair, *Potri.006G161200*–*Potri.006G161400*, was found in *Populus trichocarpa*, which showed high sequence similarity to the *Arabidopsis IAA27* gene [64]. Additionally, 13 gene pairs, largely belonging to the *IAA8* and *IAA9* subfamilies, were detected in turnip plants. Their Ka/Ks values were higher than 1, indicating that they experienced tachytelic evolution in the recent past [20]. Alongside dicots, gene pairs with Ka/Ks values of >1 were also found in gymnosperm, an example of which is *MA_10430843g0010*-*MA_10430843g0020* in *Picea abies*, which is homologous with *AtIAA14* [64].

Four characteristic conserved domains underlie the typical Aux/IAA protein functions [10]. In this study, analyses of conserved structural domains showed that some members are atypical, lacking one or more conserved domains in *Fragaria* (Table 1). For instance, subfamilies IAA14 and IAA29 lack domain I, which is also missing in the three PoptrIAA29 members (PoptrIAA29.1/2/3) and an *Arabidopsis* orthologue, AtIAA29 [24,72], indicating that these proteins show an inability to attract the TOPLESS and do not participate in typical auxin signal transduction. In this study, we found that IAA1 and IAA2 subfamily members lack domain II, which determines the stability of Aux/IAAs by recognizing the GWPPV sequence interacting with TIR1/AFB protein [73]. The *Arabidopsis* orthologues AtIAA20 and AtIAA31 cannot be rapidly biodegraded because they lack domain II [72,74]. Similar results have also been reported in rice and tomato plants [15,75]. Therefore, we speculated that these proteins’ half-lives in defects of domain II are much longer than the standard Aux/IAAs. In addition, IAA32s and IAA34s lack domains I and II and feature a truncated domain IV. Similarly, domains I and II seem to be disappearing from PoptrIAA34 as well as AtIAA34 [24,72]. Some 42 Aux/IAA proteins also exhibit this structure in turnip, which may interact with additional unknown components and participate in other processes such that Aux/IAA’s degradation does not require the mediation of SCF^TIR1^-dependent proteasomes [20]. Domains III and IV, which are shared with ARF proteins, facilitate homodimerization and heterodimerization with other Aux/IAA members [8,76] and for heterodimerization with ARFs to regulate the transcriptional activation of downstream genes [8,77]. Interestingly, unlike in studies of other plants (such as chinses hickory [25], bamboo [35], and *D. ofcinale* [32], etc.), all of the Aux/IAA proteins in the *Fragaria* contain domain III, with the exception of the IAA33 subfamily members, which contain only one conserved domain, domain IV. Some truncated proteins, such as FcIAA15 and FiIAA16, lack domain IV. These atypical Aux/IAAs may conduce to the varying roles of the auxin response processes, a topic that requires further study.

Most *aux/iaa* mutations in *Arabidopsis* reveal their functional roles during plant growth and development processes. For example, *auxin-resistant 5* (*axr5*), with a mutation in *Aux*/*IAA1*, is resistant to auxin and induces various auxin-related growth defects, including defects in tropism of roots and shoots [78]. *iaa2* and *iaa6* show similar phenotypes, and accelerate tuberous root development of *Tetrastigma hemsleyanum* by regulating hormone biosynthesis [79]. Similarly, *FveIAA2* shows higher expression in roots. *FveIAA2* and *FveIAA6* are more pronounced in the wall development process (Figure 5). The wall is a part of the achanes, which cause dotting on the receptacle surface of true strawberry fruit and are essential for the enlargement and ripening of the strawberry receptacle [39,80]. Recent studies indicate that *MdAux*/*IAA2* negatively regulates apple fruit and size of cells, and its expression is enhanced in the absence of auxin, conversely leading to small fruit size in Longfeng apple plants [81]. Two *IAA15* members (*FveIAA15a* and *FveIAA15b*) were identified in *F. vesca*. However, their promoter structure and expression patterns differed greatly (Figure 5 and Figure S5). For instance, compared with *FveIAA15a*, most ABA- and MeJA-responsive elements were located in the *FveIAA15b* promoter and *FveIAA15a* was solely expressed in the ghost4 and SAM tissues. The same was true of the two *IAA29* members (*FveIAA29a* and *FveIAA29b*). IAA29 competitively interacts with WRKY57 to confront the leaf senescence induced by MeJA [82]. In the strawberries, we found that *FveWRKY57a* exhibited a leaf-specific expression (Figure 6D), and most members of the *IAA29* subfamilies contained many MeJA-responsive elements in their promoters (Figure 3). Hence, we inferred that *FveIAA29a* and *FveWRKY57a* may be important for the MeJA-induced leaf senescence of strawberry plants. High levels of *FveIAA9* were observed in all tissues, but predominantly expressed in flower tissues. This suggested that *IAA9* may be connected to the development of flowers. In tomato, *SiIAA9* controls multiple processes mediated by auxin signaling, including apical dominance and the development of flower organs and fruit development [83]. In addition, the IAA33 subfamily has only one conserved domain and contains the majority of endosperm development-related cis-elements in strawberries. We suggest that these proteins may participate in embryo and fruit development. Overall, analysis of the structure, evolution, and expression levels of the *Aux*/*IAA* family offers an initial insight into the function of these genes in strawberry genus. These results need to be further verified in future investigation.

## 4. Materials and Methods

### 4.1. Identification and Characterization of Aux/IAA Genes in Fragaria

The genome information and related annotation files of six diploid strawberries—*F. iinumae*, *F. mandshurica*, *F. nilgerrensis*, *F. nipponica*, *F. vesca* (v4.0.a2), and *F. viridis*—and two octoploid strawberries—*F. × ananassa* (v1.0.a2) and *F. chiloensis*—were downloaded from the Genome Database for Rosaceae (https://www.rosaceae.org/) (accessed on 18 January 2024) [84]. The AtIAA protein sequences were obtained from the TAIR (https://www.arabidopsis.org/) (accessed on 18 January 2024). Twenty-nine *Aux*/*IAA* genes of *A. thaliana* were used as query sequences, and BLAST v2.10.0 [50] (E-value 1 × 10^−10^) searches were performed to obtain orthologous genes in *Fragaria* genus. The Aux/IAA domain’s hidden Markov model (HMM) (PF02309) was downloaded [52] from the Pfam databas, and HMMER v3.2.1 [51] (E-value 1 × 10^−10^) was used to search sequences with this model in the *Fragaria* genome. Finally, the shared sequences obtained by the two search tools served as candidate *Aux*/*IAA* gene family members of the strawberries. The Pfam database [52] and the Conserved Domain Database in NCBI [53] were used to identify the conserved domains. All *Fragaria* Aux/IAA protein’s physical and chemical parameters were calculated with the Expasy-ProtParam tool (https://web.expasy.org/protparam/) (accessed on 19 April 2024).

### 4.2. Phylogenetic and Duplication Analysis

The protein sequences of Aux/IAA were aligned using MEGA 11 (https://www.megasoftware.net/) (accessed on 29 April 2024) [55]. Evolutionary trees were constructed using the maximum-likelihood method with the JTT + G model (Jones–Taylor–Thornton model with a gamma distribution for among-site rate variation, bootstrap test replicates = 1000 times). Tbtools-II (v2.110) was used to predict segmental duplications and tandem duplications [56]. The synonymous (Ks) and non-synonymous (Ka) substitution rates for gene pairs were calculated by KaKs_Calculator v2.0 [57].

### 4.3. Analysis of Motif and Cis-Regulatory Elements

The identification of motifs was achieved using MEME Suite [85]. The analysis of cis-regulatory elements was conducted using the 2000 bp upstream of *Aux*/*IAA* genes using the PlantCARE website (http://bioinformatics.psb.ugent.be/webtools/Plantcare/html/) (accessed on 9 May 2024), and the subsequent results were illustrated using Tbtools-II [56].

### 4.4. Analysis of Tissue Expression Patterns in F. vesca

We obtained the expression data in diploid strawberry tissues based on an annotation file (“Expression patterns of all the genes in v4.0.a2 across diverse tissue types”) of the *F. vesca* v4.0.a2 genome [58]. For anther and carpel tissues, 7, 8, 9, 10, 11, and 12 represent the developmental stages of *F. vesca* flower. For the other tissues (including style, ovule, embryo, ghost, seed, wall, cortex, and pith), 1, 2, 3, 4, and 5 represent the developmental stages of *F. vesca* fruit.

### 4.5. PPI Network Prediction

The PPI network was established based on the STRING website (http://cn.string-db.org) (accessed on 7 August 2024). Then, a BLASTP search of the *F. vesca* protein database [58] was performed, with the aim of finding the hub proteins of homologous *A. thaliana*, as predicted using the STRING website.

## 5. Conclusions

A total of 287 *Aux*/*IAA*s were identified in eight strawberry species in this work. Analyses of the sequence structures (including motifs, conserved domains, and promoter) revealed the characterization of *Aux*/*IAA*s. In addition, evolutionary trees and collinearity analyses revealed that segmental duplications drove *Aux*/*IAA* evolution in *Fragaria* species, which was followed by purifying selection. Moreover, the tissue expression profiles of *Aux*/*IAA*s showed that most genes potentially participated in the development of seeds and fruits in strawberries. In short, this study not only provides a foundation for the functions of *Aux/IAAs* but also offers proof for the evolution of strawberries.

## Figures and Tables

**Table 1:** The completeness of Aux/IAAs in *Arabidopsis* and *Fragaria*.

Species	Protein No.	Truncated Proteins	Complete Proteins
*Arabidopsis thaliana*	29	11 (38%)	18 (62%)
*Fragaria x ananassa*	82	36 (43.9%)	46 (56.1%)
*Fragaria chiloensis*	75	33 (44%)	42 (56%)
*Fragaria iinumae*	18	7 (38.9%)	11 (61.1%)
*Fragaria mandshurica*	22	9 (40.9%)	13 (59.1%)
*Fragaria nilgerrensis*	20	8 (40%)	12 (60%)
*Fragaria nipponica*	27	9 (33.3%)	18 (66.7%)
*Fragaria vesca*	22	8 (36.4%)	14 (63.6%)
*Fragaria viridis*	21	7 (33.3%)	14 (66.7%)

## Data Availability

The data presented in this study are available in the article and Supplementary Materials.

## Supplementary Materials

The following supporting information can be downloaded at https://www.mdpi.com/article/10.3390/plants13202940/s1. Figure S1: Motif analysis of Aux/IAA protein family in *F. chiloensis*; Figure S2: Motif analysis in Aux/IAA proteins of six diploid strawberry species; Figure S3: The results of Ka, Ks, and Ka/Ks values of gene pairs in six *Fragaria* species; Figure S4: Ka/Ks > 1 of gene pairs in eight strawberry species; Figure S5: Promoter cis-acting elements predicted in *Aux*/*IAA*s from *F. vesca*; Table S1: Basic information of *AuxIAA* genes in eight strawberry species; Table S2: Physical and chemical coefficients of *Fragaria* AuxIAAs; Table S3: Gene pairs exhibiting Ka/Ks > 1 in eight strawberry species.
