# Supplementary figures and images for: Genome-Wide Identification and Characterization of the Aux/IAA Gene Family in Strawberry Species
Source: Plants (Basel). 2024 Oct 21;13(20):2940. doi: 10.3390/plants13202940 (PMC11511250; doi:10.3390/plants13202940)

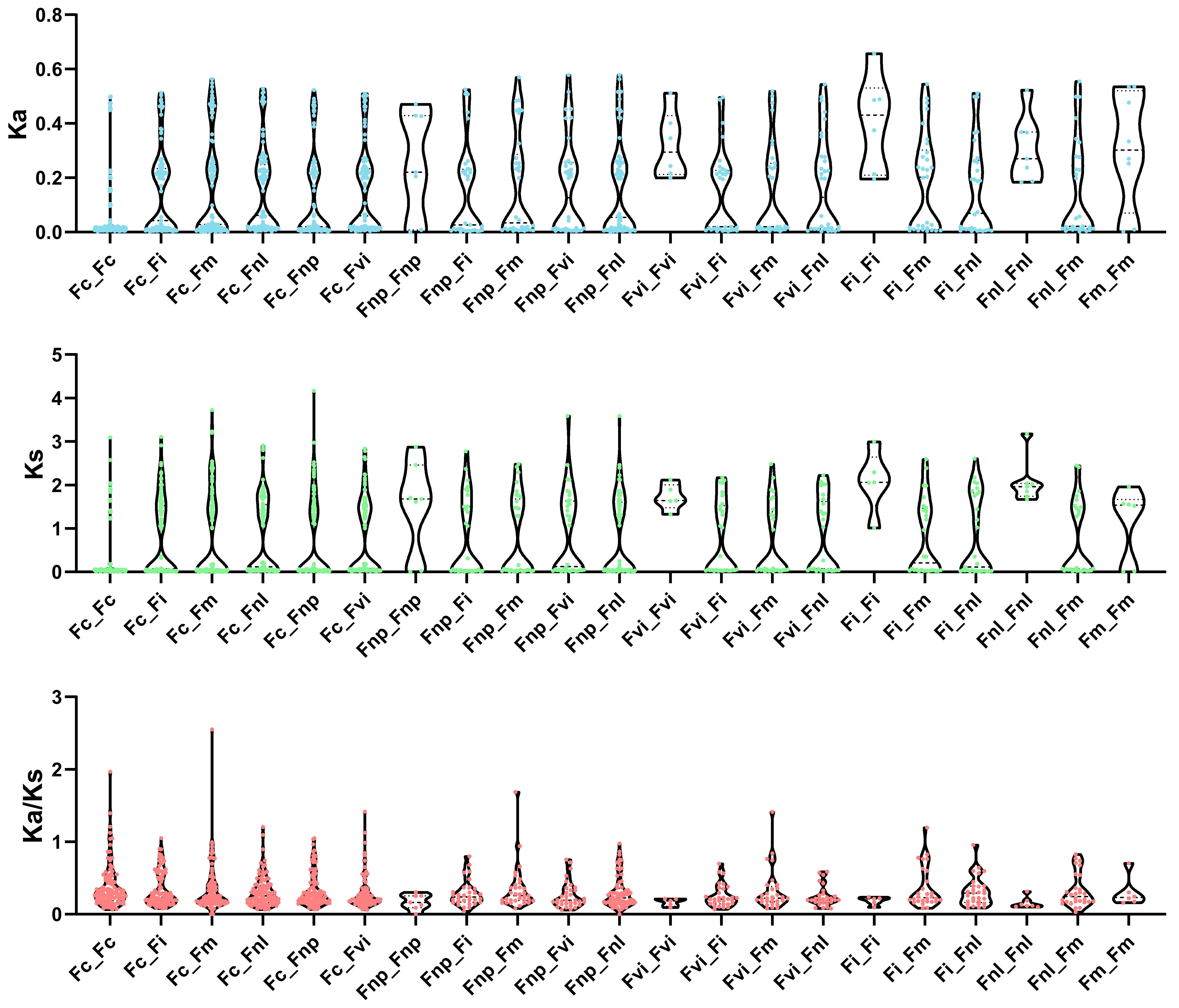

Supplement: Supplementary file 1 [file plants-13-02940-s001.zip › FigureS3.jpg]

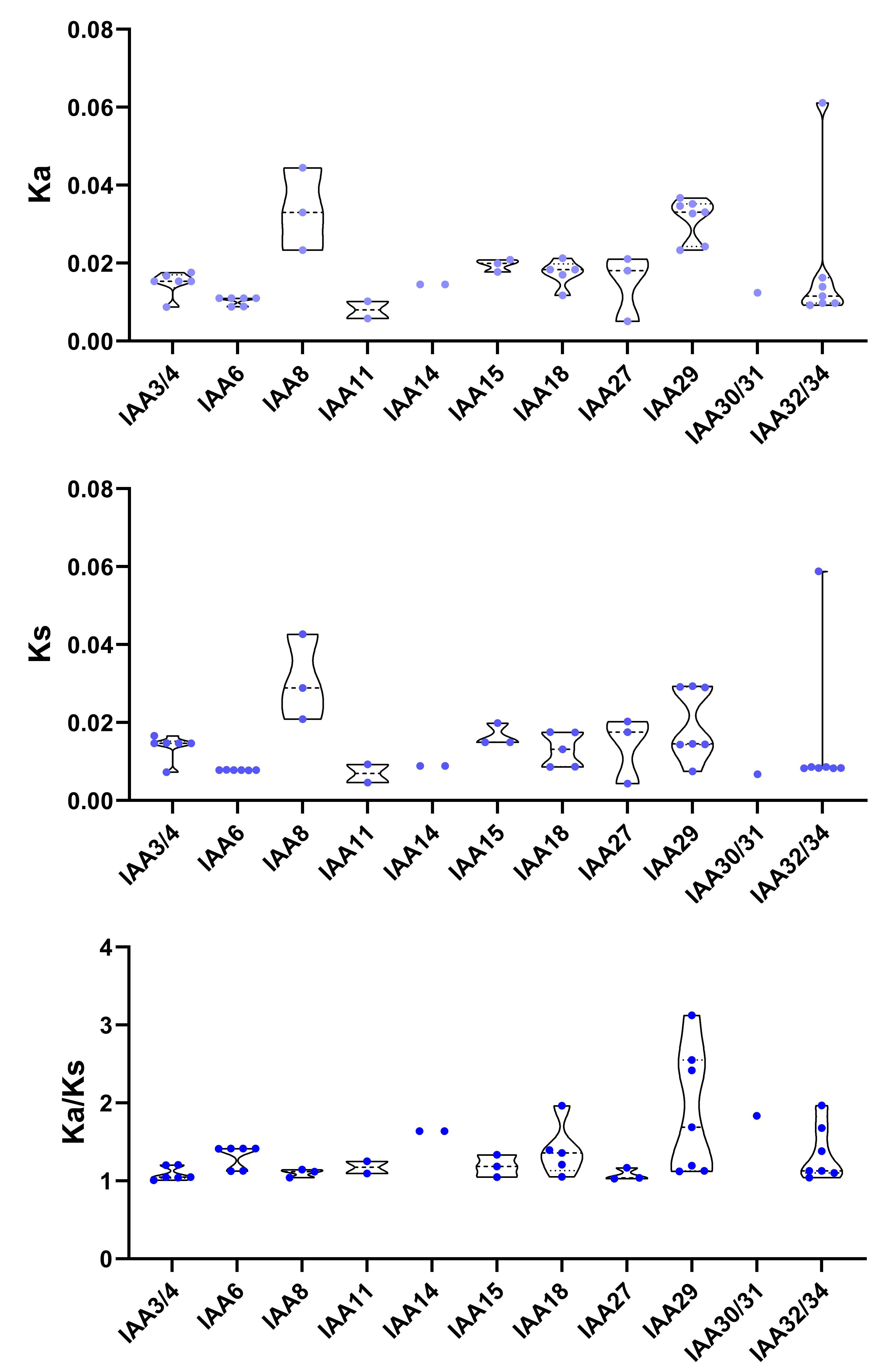

Supplement: Supplementary file 1 [file plants-13-02940-s001.zip › FigureS4.jpg]

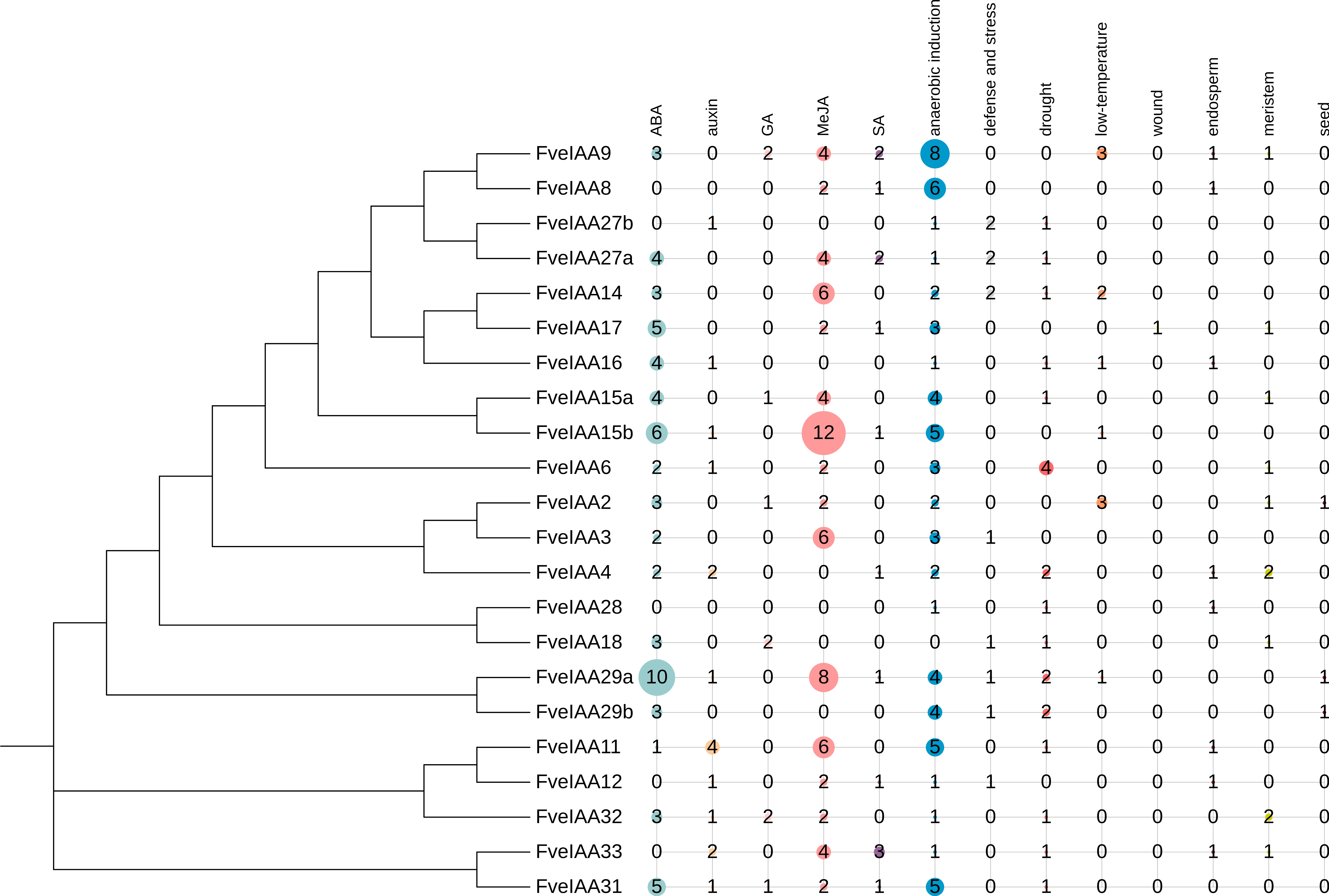

Supplement: Supplementary file 1 [file plants-13-02940-s001.zip › FigureS5.jpg]
